# Supplementary material for: Interactions between the Parasite Philasterides dicentrarchi and the Immune System of the Turbot Scophthalmus maximus. A Transcriptomic Analysis
Source: Biology (Basel). 2020 Oct 15;9(10):337. doi: 10.3390/biology9100337 (PMC7602577; doi:10.3390/biology9100337)
Supplement: Supplementary file 1 [file biology-09-00337-s001.zip › biology-939285-suppl-final/Supplementary Materials/biology-939285-suppl-proof Revised.docx]

Article

Interactions between The Parasite *Philasterides dicentrarchi* and The Immune System of The Turbot *Scophthalmus maximus*. A Transcriptomic Analysis

Alejandra Valle, José Manuel Leiro, Patricia Pereiro, Antonio Figueras, Beatriz Novoa, Ron P. H. Dirks and Jesús Lamas

**Supplementary Materials**

| (**A**) | 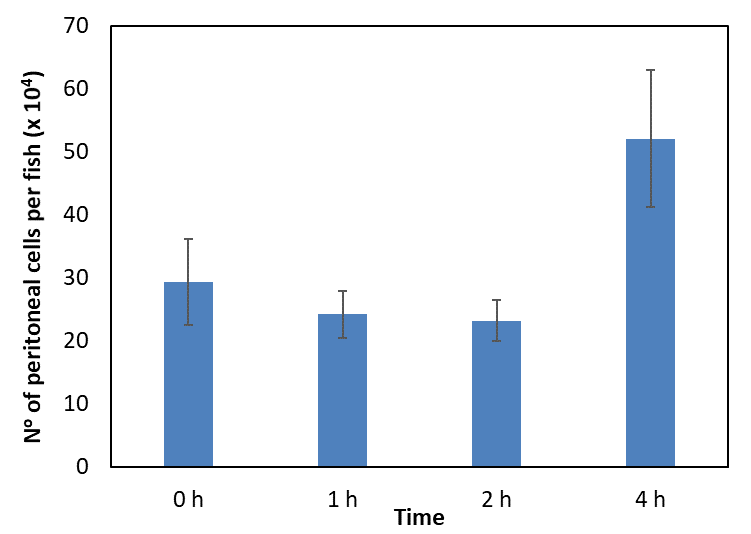 * |
| --- | --- |
| (**B**) |  |

**Figure S1.** Number of turbot cells and ciliates in the peritoneal cavity. Estimated numbers of free peritoneal cells (A) and ciliates (B) in the peritoneal cavity of infected turbot at 0, 1, 2, and 4 hpi. Values represent the mean ± SD. **p* < 0.05 relative to 0 h.

**
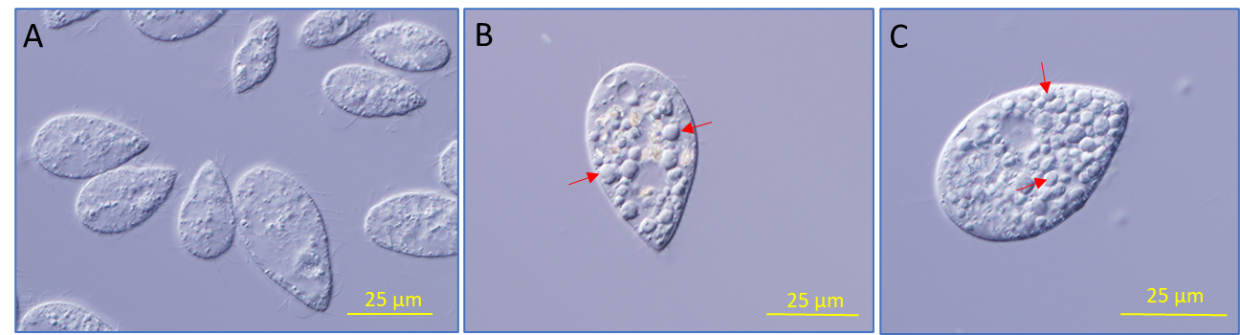
Figure S2.** Micrographs of ciliates at different stages of infection. Differential interference contrast microscopy micrographs showing the ciliates at 0 (A), 1 (B) and 4 (C) hpi. Note that ciliates contain numerous phagocytosed leukocytes (red arrows) at 1 and 4 hpi.

| (A) |  |
| --- | --- |
| (B) |  |

**Figure S3.** qPCR validation of RNA-seq findings**.** Expression profile of selected genes in *P. dicentrarchi* (**A**) and in turbot peritoneal cells (**B**). Gene expression was normalized using *P. dicentrarchi* and turbot *ef1-α* as the reference genes. Results are expressed as the normalized expression in experimental groups, divided by the normalized expression in the control group. *P. dicentrarchi* genes included: *ctsb*633 (*cathepsin B*, contig 633), *lmln*1589 (*leishmanolysin*, contig 1589), *lmnl*17670 (*leishmanolysin*, contig 17670), *abcg10* (*ABC transporter G family member 10*), and *abcg11* (*ABC transporter G family member 11*). Turbot genes included: *tnfa* (*tumor necrosis factor alpha*), *il1b* (*interleukin 1 beta*), *il8* (*interleukin 8*), *stat6* (*signal transducer and activator of transcription 6*), and *cd11b*.

**Table S1.** Sample information. Eight samples were analysed (in triplicate), including four samples of ciliates and peritoneal fish cells obtained at 0, 1, 2 and 4 hours post injection (Ph1-Ph4) and four samples of peritoneal cells obtained from fish injected with PBS (PBS 12, 48) or ciliates (CIL 12, 48) and sampled at 12 or 48 hpi.

| **ZFS Code** | **Lamas Code** | **Description** |
| --- | --- | --- |
| ZFG-16-14_1 | Ph 0-1 | Ciliates 0 hours, replicate 1 |
| ZFG-16-14_2 | Ph 0-2 | Ciliates 0 hours, replicate 2 |
| ZFG-16-14_3 | Ph 0-3 | Ciliates 0 hours, replicate 3 |
| ZFG-16-14_4 | Ph 1-1 | Ciliates 1 hours, replicate 1 |
| ZFG-16-14_5 | Ph 1-2 | Ciliates 1 hours, replicate 2 |
| ZFG-16-14_6 | Ph 1-3 | Ciliates 1 hours, replicate 3 |
| ZFG-16-14_7 | Ph 2-1 | Ciliates 2 hours, replicate 1 |
| ZFG-16-14_8 | Ph 2-2 | Ciliates 2 hours, replicate 2 |
| ZFG-16-14_9 | Ph 2-3 | Ciliates 2 hours, replicate 3 |
| ZFG-16-14_10 | Ph 4-1 | Ciliates 4 hours, replicate 1 |
| ZFG-16-14_11 | Ph 4-2 | Ciliates 4 hours, replicate 2 |
| ZFG-16-14_12 | Ph 4-3 | Ciliates 4 hours, replicate 3 |
| ZFG-16-14_13 | PBS 12-1 | Fish cells, PBS 12 hours, replicate 1 |
| ZFG-16-14_14 | PBS 12-2 | Fish cells, PBS 12 hours, replicate 2 |
| ZFG-16-14-15 | PBS 12-3 | Fish cells, PBS 12 hours, replicate 3 |
| ZFG-16-14_16 | CIL 12-1 | Fish cells, Infected 12 hours, replicate 1 |
| ZFG-16-14_17 | CIL 12-2 | Fish cells, Infected 12 hours, replicate 2 |
| ZFG-16-14_18 | CIL 12-3 | Fish cells, Infected 12 hours, replicate 3 |
| ZFG-16-14_19 | PBS 48-1 | Fish cells, PBS 48 hours, replicate 1 |
| ZFG-16-14_20 | PBS 48-2 | Fish cells, PBS 48 hours, replicate 2 |
| ZFG-16-14_21 | PBS 48-3 | Fish cells, PBS 48 hours, replicate 3 |
| ZFG-16-14_22 | CIL 48-1 | Fish cells, Infected 48 hours, replicate 1 |
| ZFG-16-14_23 | CIL 48-2 | Fish cells, Infected 48 hours, replicate 2 |
| ZFG-16-14_24 | CIL 48-3 | Fish cells, Infected 48 hours, replicate 3 |

**Table S2.** Primer sequences used in the qPCR analysis.

| **Gene Name** | **Forward Primer (5′→3′)** | **Reverse pPrimer(5′→3′)** |
| --- | --- | --- |
| *β-Actin* (*P. dicentrarchi*) | F: TGTTCTTCGGGAGCAACTCT | R: CCGGTATTATGGTCGGAATG |
| *Elongation factor 1-alpha*  (*P. dicentrarchi*) | F: TCGCTCCTTCTTGCATCGTT | R: TCTGGCTGGGTCGTTTTTGT |
| *Cathepsin B* | F: ACCAACGGACCTGTTACTGC | R: TAATCCTCCGAGACCCCAGT |
| *Leishmanolysin -like peptidase 1589* | F: AGATGCTGGAAAGGTTGTGC | R: ATGGAATCAGCAGGACAAGG |
| *Leishmanolysin -like peptidase 17670* | F: TTTCCGAACCAGTTTTCAGG | R: CAAAGACGGCCAATGTTCTT |
| *ABC transporter G family member 10* | F: CCTTCTGGAGGAGGGAAAAC | R: GCAGCGTAATCAAGGGTCTC |
| *ABC transporter G family member 11* | F: ATTTTTGGATGAACCCACCA | R: CCGAAATAGGCAGTGTTTCC |
| *β-actin* (turbot) | F: TTTGAGCAGGAAATGGGAAC | R: GTGTTGGCGTACAGGTCCTT |
| *Elongation factor 1-alpha* (turbot) | F: GGAGGCCAGCTCAAAGATGG | R: ACAGTTCCAATACCGCCGATTT |
| *glyceraldehyde-3-phosphate dehydrogenase* | F: TCCAATGTTTGTCATGGGAGTT | R: CCAGAGGAGCCAGGCAGTT |
| *tumor necrosis factor alpha* | F: CCCTTATCATTATGGCCCTT | R: TCCGAGTACCGCCATATCCT |
| *cd11b* | F: AGGTTCATGGGAAGACTGGA | R: ATTGGACCCTGCTGAAAAGA |
| *stat6* | F: TCAAGACAACAACGCTCTGG | R: CTGTGTCTGGACCTCAGCAA |
| *c-x-c motif chemokine ligand 8* | F CCATCAGTGAAGGGGTGAGT | R: GAGTGTTTCAGGGTGGCAAT |
| *interleukin 1 beta* | F: CACACAAACACAACTCCTCCTC | R: TGTTGCTCCACATCTCGCTC |

| 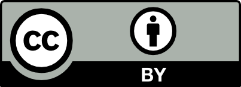 | © 2020 by the authors. Licensee MDPI, Basel, Switzerland. This article is an open access article distributed under the terms and conditions of the Creative Commons Attribution (CC BY) license (http://creativecommons.org/licenses/by/4.0/). |
| --- | --- |
